# Breast Cancer Metastasis to the Thyroid Presenting as a Collision Tumor With Papillary Thyroid Microcarcinoma and Oncocytic Adenoma: A Case Report

**DOI:** 10.7759/cureus.117210

**Published:** 2026-09-30

**Authors:** Diego G Lanza Botia, Rafael E Tejada, Julian A Tellez Villa, Jinneth Acosta, Fabian G Barrero Florez, Jennifer Parra Sanchez

**Affiliations:** 1 Department of Internal Medicine, Universidad Nacional de Colombia, Bogota, COL; 2 Department of Internal Medicine, Hospital Universitario Nacional de Colombia, Bogota, COL; 3 Department of Hematology and Oncology, Hospital Universitario Nacional de Colombia, Bogota, COL; 4 Department of Pathology, Hospital Universitario Nacional de Colombia, Bogota, COL; 5 Department of Pathology, Universidad Nacional de Colombia, Bogota, COL; 6 Department of Epidemiology, Fundación Universitaria de Ciencias de la Salud, Bogota, COL

**Keywords:** breast cancer metastasis, collision tumor, oncocytic adenoma, papillary thyroid microcarcinoma, thyroid metastasis

## Abstract

Thyroid metastases from breast cancer are uncommon, and their coexistence with primary thyroid neoplasms as collision tumors is even rarer, posing important diagnostic and therapeutic challenges. We report the case of a 57-year-old woman with hormone receptor-positive, HER2-negative breast cancer who developed disease recurrence 13 years after initial treatment, presenting with a cervical mass, persistent dry cough, and dysphagia. Evaluation revealed an intermediate-suspicion thyroid nodule according to the American Thyroid Association (ATA) classification, with successive fine-needle aspiration biopsies categorized as Bethesda III and IV. Right hemithyroidectomy demonstrated poorly differentiated metastatic breast carcinoma, confirmed by immunohistochemistry, coexisting within the same thyroid specimen with a 1.8-mm papillary thyroid microcarcinoma (PTMC) and a 7-mm oncocytic adenoma, constituting an exceptionally rare thyroid collision tumor. Staging studies subsequently revealed systemic metastatic disease, and treatment with ribociclib plus fulvestrant was initiated according to the phase III MONALEESA-3 regimen. This case highlights the possibility of very late recurrence of hormone receptor-positive breast cancer presenting as thyroid metastasis, illustrates the diagnostic complexity of thyroid collision tumors, and emphasizes the importance of considering metastatic disease in the differential diagnosis of thyroid nodules in patients with a history of breast cancer. Although current evidence does not support routine thyroid screening, this case raises the question of whether targeted thyroid evaluation in selected patients at high risk of late recurrence warrants further prospective investigation.

## Introduction

Metastases to the thyroid from breast carcinoma are uncommon. Thyroid surgical series show metastases from non-thyroidal primary tumors in a small percentage of cases, ranging from 1.4% to 3%, of which breast origin accounts for 7.8% [1]. Additionally, a bidirectional relationship between breast cancer and thyroid cancer has been reported, with these tumors more frequently occurring as metachronous tumors; however, current evidence regarding risk factors for their development remains scarce [2].

Hormone receptor-positive breast cancer is characterized by the possibility of developing disease recurrence several years after initial remission, with studies documenting an almost linear relationship between recurrence risk and time elapsed since diagnosis [3], as well as more than 50% of relapses occurring after five years [4]. Given this, National Comprehensive Cancer Network (NCCN) guidelines recommend prolonged clinical follow-up with physical examination and annual mammography indefinitely [5].

We present the case of a 57-year-old patient with recurrence of hormone receptor-positive breast cancer 13 years after diagnosis, manifesting as a solid thyroid nodule, with subsequent documentation of thyroid metastases from breast cancer, as well as a papillary thyroid microcarcinoma (PTMC) and an oncocytic adenoma in the same surgical specimen. 

The finding of these tumors in the same specimen has been termed a “collision tumor,” a rare neoplastic entity defined as the presence of two or more histologically distinct tumors coexisting at the same anatomical site while maintaining independent borders from one another [6].

In this context, the evaluation of thyroid nodules in patients with a history of breast cancer represents a diagnostic challenge. Thyroid metastases can mimic primary neoplasms both clinically and cytologically, especially in indeterminate categories of the Bethesda system, where distinguishing primary thyroid neoplasms from metastatic disease may be particularly challenging [1,7]. This difficulty increases in the presence of concomitant thyroid lesions, as occurs in collision tumors.

Published cases describing the coexistence of metastatic breast carcinoma, PTMC, and oncocytic adenoma within the same thyroid specimen are exceptionally rare. Kiziltan et al. [8] previously reported metastatic breast carcinoma involving a papillary thyroid carcinoma; however, to our knowledge, the additional coexistence of an oncocytic adenoma has not been previously described. This case highlights the diagnostic challenges of evaluating thyroid nodules in patients with a history of breast cancer, describes the pathological features of this unusual collision tumor, and discusses its implications for long-term clinical surveillance.

## Case presentation

Patient information

A 57-year-old woman had a medical history of dyslipidemia, migraine, osteoporosis, and stage IIIA, hormone receptor-positive, HER2-negative left breast cancer diagnosed 13 years prior to presentation. Initial treatment consisted of quadrantectomy with axillary lymph node dissection, adjuvant chemotherapy with the ACT regimen (doxorubicin, cyclophosphamide, and paclitaxel), and radiotherapy. She subsequently completed five years of adjuvant endocrine therapy with tamoxifen. She remained free of documented disease recurrence during routine oncologic follow-up until her presentation.

Clinical findings

At presentation (Month 0), the patient reported a sensation of a neck mass, dysphagia to solid foods, and a persistent dry cough of several months' duration that did not improve despite symptomatic treatment. Because of the persistence of these symptoms, neck ultrasonography and high-resolution computed tomography of the chest (HRCT) were performed.

Diagnostic evaluation

Thyroid ultrasonography demonstrated a 10.4 × 6.3 × 7.3-mm solid hypoechoic nodule classified as intermediate suspicion according to the American Thyroid Association (ATA) classification and TI-RADS (Thyroid Imaging Reporting and Data System) 4, as shown in Figure 1 [9, 10]. Given these findings, ultrasound-guided fine-needle aspiration (FNA) was performed at month 8 and was categorized as Bethesda III. Repeat FNA at month 14 yielded a Bethesda IV result, prompting referral for right hemithyroidectomy [7].

**Figure 1:**
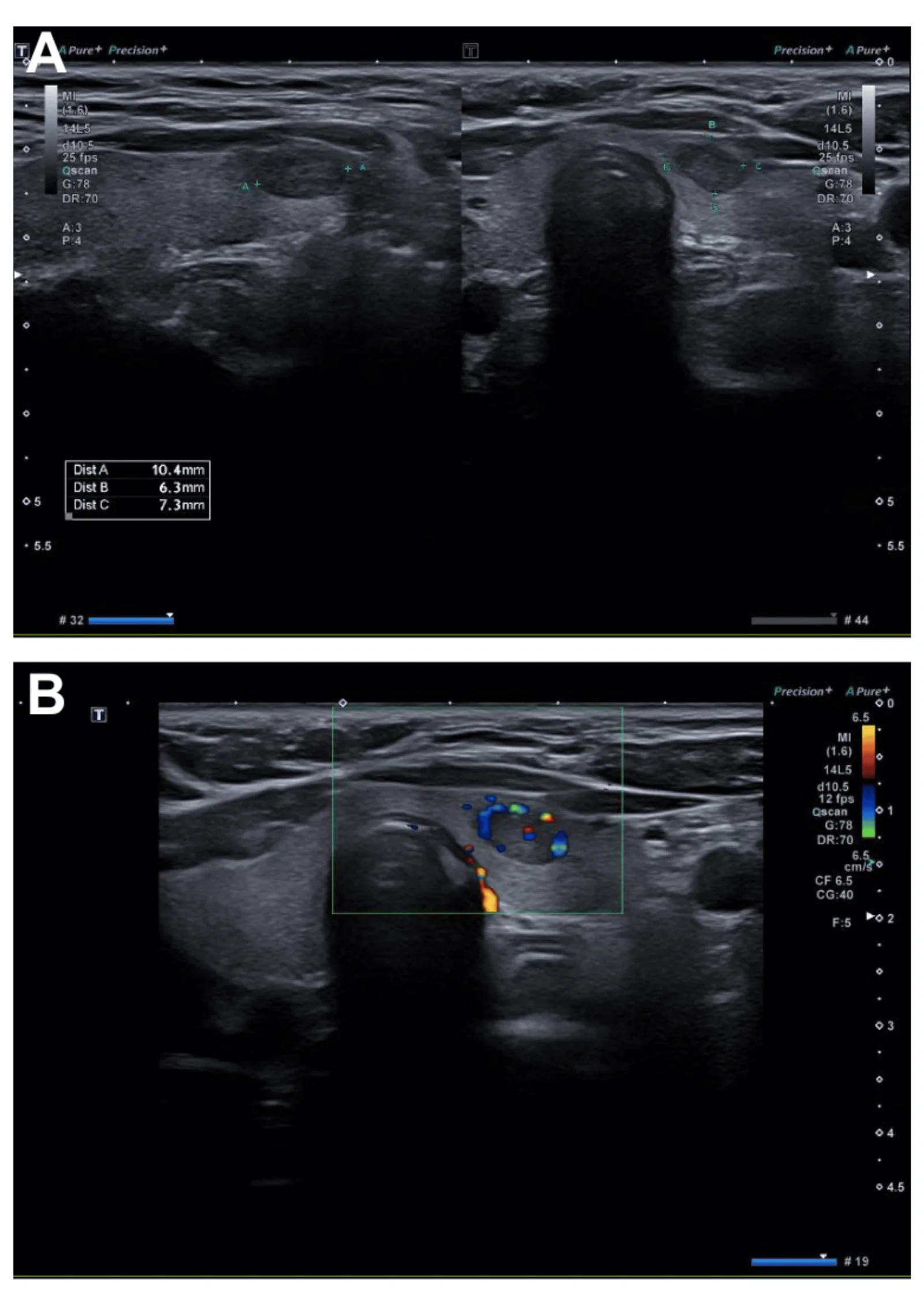
Thyroid ultrasonography showing an intermediate-suspicion thyroid nodule according to the American Thyroid Association classification (A) Gray-scale ultrasonography demonstrates a 10.4 × 6.3 × 7.3 mm solid, hypoechoic nodule located at the junction of the thyroid isthmus and the left lobe that is wider than tall, with smooth margins and without comet-tail artifacts or internal calcifications. (B) Color Doppler ultrasonography demonstrates peripheral and intranodular vascularization. Reference: [9]

Given the Bethesda IV cytology findings, the patient was evaluated by the head and neck surgery service, and right hemithyroidectomy was recommended [7]. Because of the complexity of the pathological findings, the case was subsequently reviewed at a multidisciplinary pathology conference. Final histopathological examination demonstrated three distinct neoplastic lesions within the same thyroid specimen: a 1.8-mm PTMC, a 7-mm oncocytic adenoma, and a 4.2-mm lesion corresponding to metastatic poorly differentiated breast carcinoma with a hormone receptor-positive, HER2-negative immunophenotype and a Ki-67 proliferation index of 30% (Figures 2, 3). Immunohistochemical staining confirmed the metastatic breast origin of the lesion, supporting the final diagnosis of a thyroid collision tumor.

**Figure 2:**
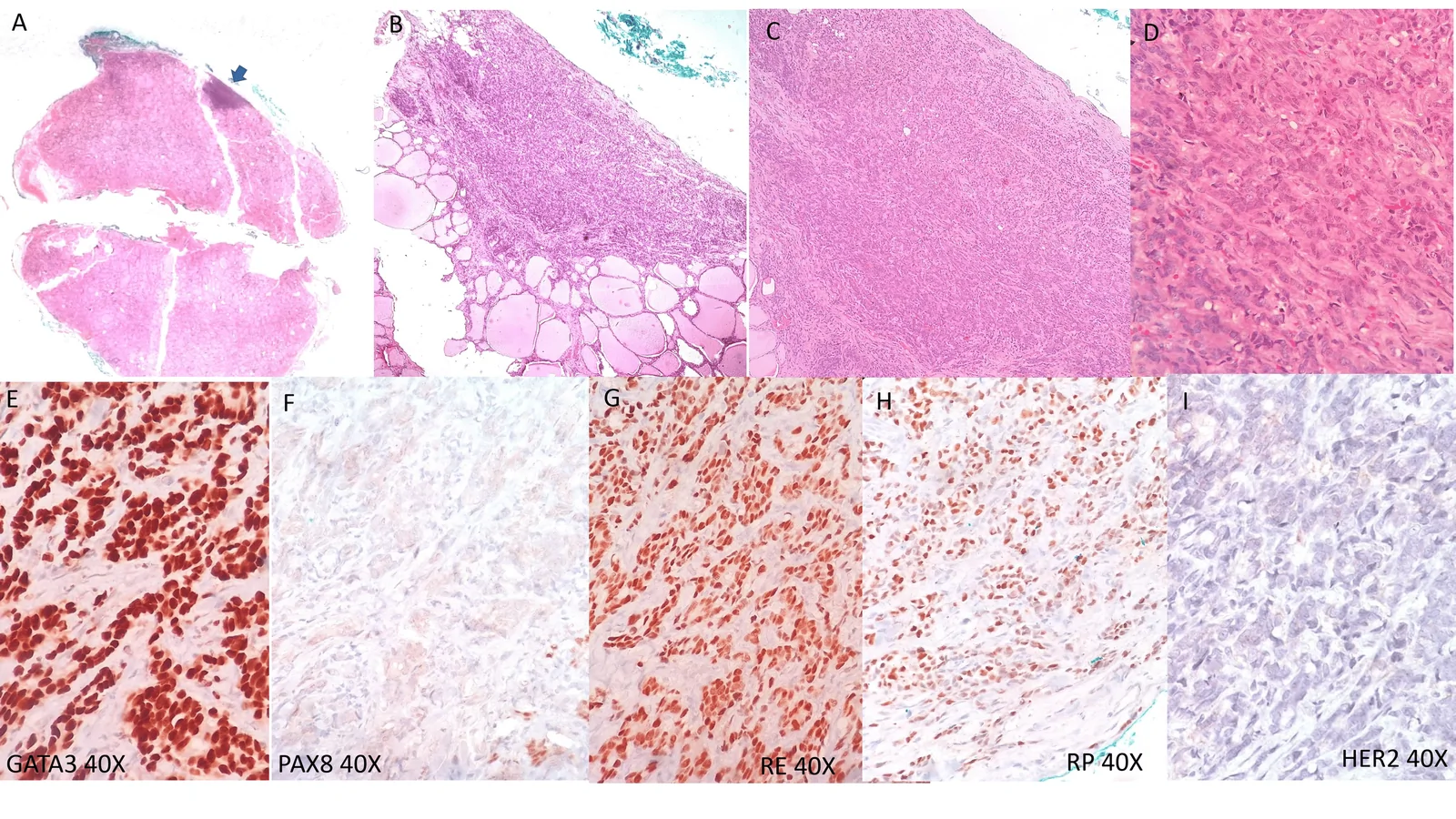
Histopathological and immunohistochemical findings of metastatic breast carcinoma involving the thyroid. A–D: Hematoxylin and eosin (H&E) staining at ×1, ×10, ×20, and ×40 magnification, respectively. The arrow in (A) highlights a superficial 4.2-mm lesion corresponding to metastatic poorly differentiated breast carcinoma. (E–I): Immunohistochemical staining at ×40 magnification for GATA3, PAX8, estrogen receptor (ER), progesterone receptor (PR), and HER2, respectively. The metastatic tumor cells demonstrate nuclear expression of GATA3 and absence of PAX8 expression, while residual thyroid tissue shows nuclear PAX8 expression. The tumor cells show expression of ER and PR, with no membranous immunoreactivity for HER2.

**Figure 3:**
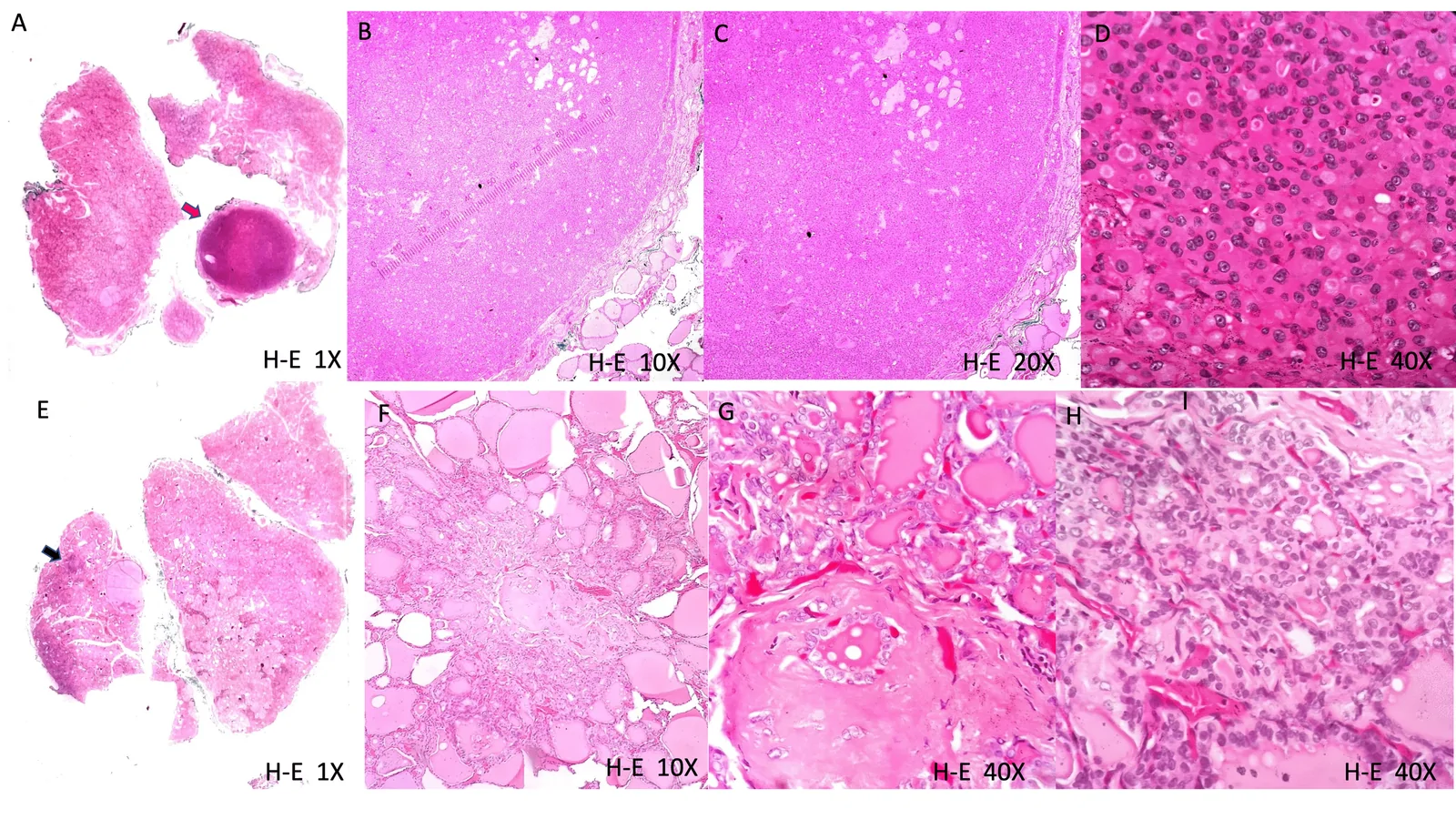
Histopathological findings of oncocytic adenoma and papillary thyroid microcarcinoma. (A–D) Hematoxylin and eosin (H&E) staining at ×1, ×10, ×20, and ×40 magnification, respectively. The arrow in (A) highlights an encapsulated 7-mm oncocytic lesion corresponding to an oncocytic adenoma. Images (B–D) highlight its cellular characteristics and oncocytic appearance. (E–H) H&E staining at ×1, ×10, ×40, and ×40 magnification, respectively. The arrow in (E) highlights a 1.8-mm nodule corresponding to classic papillary thyroid microcarcinoma. Images (F–H) highlight the characteristic papillary architecture and nuclear features of papillary thyroid carcinoma.

Meanwhile, the patient had been followed for approximately one year before presentation because of an occasional dry cough and exertional dyspnea, despite normal pulmonary function test results. HRCT of the chest identified pulmonary nodules measuring 4.8 mm in the left lower lobe and 1.8 mm in the left pulmonary apex. At presentation, follow-up HRCT demonstrated a new 4-mm nodule in the right lower lobe. By month 12 after presentation, this nodule had increased to 6 mm. By month 23, HRCT demonstrated a marked increase in the number and size of bilateral pulmonary nodules, with the largest measuring 9 mm, prompting pulmonary segmentectomy for diagnostic confirmation. Histopathological examination of the resected nodules confirmed metastatic breast carcinoma. The chronological evolution of the pulmonary nodules is illustrated in Figure 4. These findings established systemic recurrence of hormone receptor-positive breast cancer, with metastatic involvement of the thyroid and lungs.

**Figure 4:**
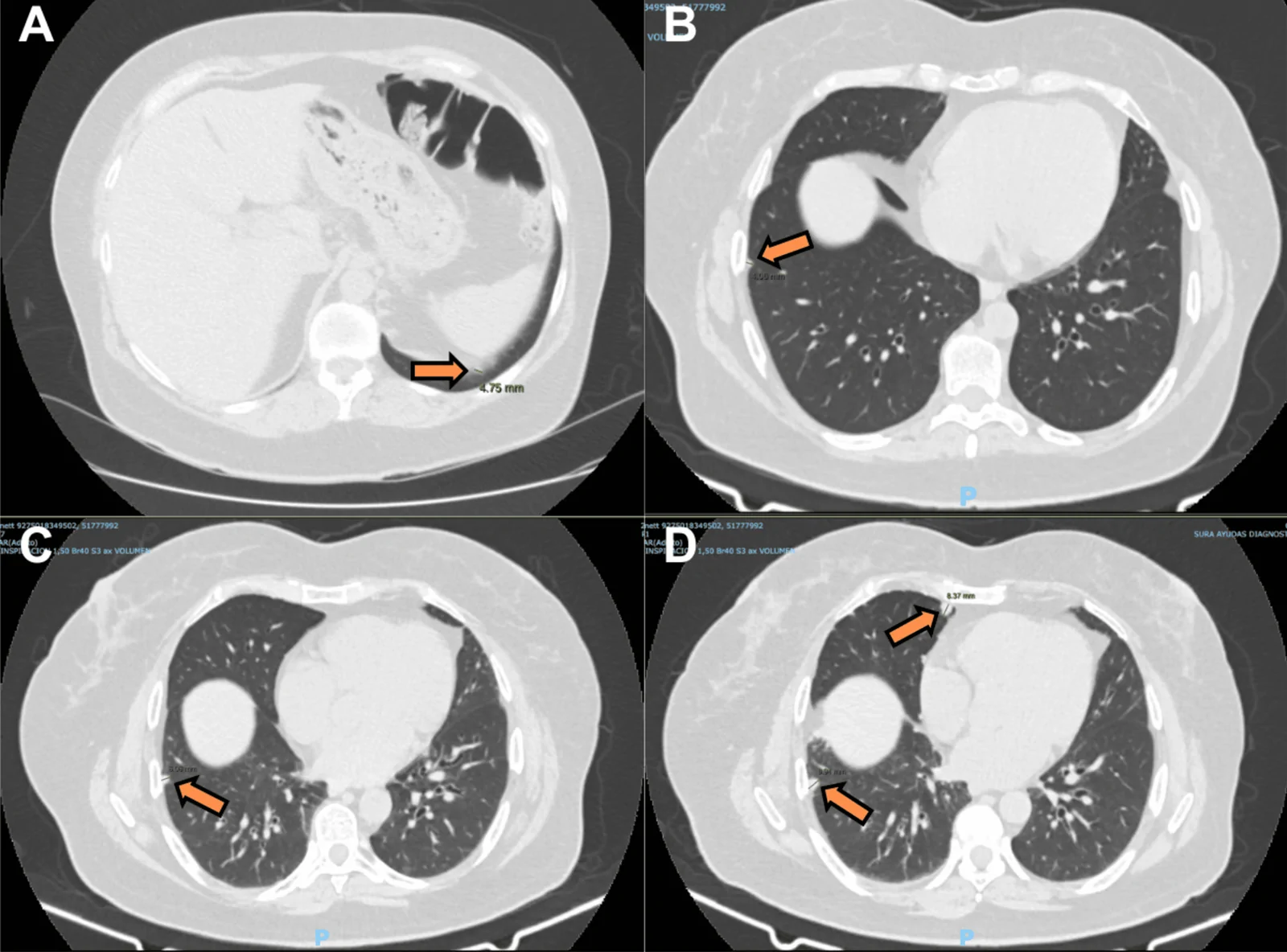
Serial high-resolution computed tomography (HRCT) of the chest demonstrating the chronological evolution of the patient's pulmonary nodules. (A) Approximately one year before presentation: solid pulmonary nodules measuring 4.8 mm in the left lower lobe and 1.8 mm in the left pulmonary apex. (B) Month 0: a new 4-mm nodule in the right lower lobe, with the previously identified nodules remaining stable. (C) Month 12: enlargement of the right lower lobe nodule to 6 mm. (D) Month 23: increase in the number and size of bilateral pulmonary nodules, with the largest nodule, located in the right lower lobe, measuring 9 mm. The arrows in (A–D) highlight the pulmonary nodules described in the text.

Following histopathological confirmation of metastatic breast carcinoma involving both the thyroid and pulmonary nodules, the patient was referred to the Clinical Oncology service for complete staging and evaluation. Contrast-enhanced computed tomography of the chest and abdomen, positron emission tomography/computed tomography (PET/CT), and bone scintigraphy demonstrated widespread osseous metastatic disease involving the skull, cervical, thoracic, and lumbar spine, sternum, ribs, and sacrum.

The patient was diagnosed with systemic recurrence of hormone receptor-positive, HER2-negative breast cancer, together with a surgically treated PTMC and a benign oncocytic adenoma. The patient's prognosis was considered to be driven by the metastatic breast carcinoma. Next-generation sequencing (NGS) identified no clinically actionable genomic alterations.

Therapeutic intervention

Given the diagnosis of hormone receptor-positive, HER2-negative metastatic breast cancer, systemic therapy was initiated in month 26, with ribociclib plus fulvestrant according to the phase III MONALEESA-3 regimen [11]. Ribociclib was administered at the standard dose of 600 mg daily (21 days on/7 days off), together with fulvestrant 500 mg according to the approved dosing schedule. Because of extensive bone metastases, zoledronic acid (4 mg intravenously every three months) was also initiated for the prevention of skeletal-related events. Treatment was well tolerated, with no dose reductions or treatment interruptions during the reported follow-up.

Follow-up and outcomes

At the most recent follow-up, at month 50, the patient remained on systemic therapy without interruption since its initiation. Clinically, she remained stable, with no evidence of symptomatic disease progression. The most recent imaging assessment demonstrated a partial radiological response according to Response Evaluation Criteria in Solid Tumors, version 1.1 (RECIST 1.1) criteria [12], with no evidence of disease progression.

Treatment-related adverse effects were limited to mild asthenia and occasional headache during the first days of each treatment cycle, neither of which required dose reduction or treatment interruption. During follow-up, complete blood counts and liver function tests were monitored, and electrocardiograms were performed for cardiac safety monitoring. No clinically significant hematologic, hepatic, or cardiac abnormalities requiring treatment interruption or dose reduction were documented. These symptoms did not interfere with her activities of daily living, and she remained functionally independent throughout follow-up. The patient reported satisfaction with the current treatment because of symptom control and her ability to maintain her usual daily activities.

The patient's diagnostic evaluation and therapeutic course are summarized in Table 1.

**Table 1:** Diagnostic and therapeutic course HR: hormone receptor; ACT: doxorubicin/cyclophosphamide/paclitaxel; Bethesda: The Bethesda System for Reporting Thyroid Cytopathology; HRCT: high-resolution computed axial tomography; TI-RADS: Thyroid Imaging Reporting and Data System; FNA: fine-needle aspiration biopsy; Ki-67: cell proliferation marker; MONALEESA-3: phase III clinical trial evaluating ribociclib plus fulvestrant in hormone receptor-positive, HER2-negative advanced breast cancer; RECIST 1.1: Response Evaluation Criteria in Solid Tumors, version 1.1.

Time	Diagnosis and treatment
~ 13 to 12 years before presentation	Diagnosis of hormone receptor-positive, HER2-negative breast cancer, stage IIIA. Quadrantectomy and axillary lymph node dissection. Adjuvant ACT chemotherapy. Adjuvant breast/chest wall radiotherapy.
~ 12 to seven years before presentation	Adjuvant endocrine therapy with tamoxifen for five years.
~ Seven to one year before presentation	Routine oncologic follow-up without evidence of disease recurrence.
~ One year before presentation	Persistent dry cough. Chest HRCT with nodules of 4.8 mm in the left lower basal lobe and 1.8 mm in the left pulmonary apex.
Month 0	Neck mass, dysphagia, and persistent dry cough. Thyroid ultrasound with TI-RADS 4 nodule [10]. Chest HRCT with new 4 mm nodule in the right lower basal lobe; 4.8 mm and 1.8 mm nodules stable.
Month 8	Thyroid FNA with Bethesda III diagnosis [7].
Months 12 – 14	Chest HRCT showed enlargement of the right lower lobe pulmonary nodule to 6 mm. Thyroid FNA with Bethesda IV diagnosis [7].
Months 20 – 22	Right hemithyroidectomy. Pathology demonstrated poorly differentiated metastatic breast carcinoma coexisting with a 1.8-mm papillary thyroid microcarcinoma and a 7-mm oncocytic adenoma (collision tumor).
Months 23 – 25	Chest HRCT demonstrated progression of bilateral pulmonary nodules (largest 9 mm). Pulmonary segmentectomy confirmed metastatic breast carcinoma.
Months 26 – 50	Systemic therapy with ribociclib plus fulvestrant was initiated according to the MONALEESA-3 regimen [11]. Partial radiologic response by RECIST 1.1 without progression at last follow-up [12].

## Discussion

This case illustrates several important clinical challenges in the management of patients with hormone receptor-positive breast cancer. It highlights the possibility of very late disease recurrence presenting as thyroid metastasis, an uncommon manifestation that, in this patient, coexisted with PTMC and oncocytic adenoma within the same thyroid specimen, constituting an exceptionally rare collision tumor. Although thyroid metastases account for only 1.4%-3% of thyroid malignancies in surgical series, autopsy studies have reported thyroid involvement in up to 24% of patients with disseminated malignancies, suggesting that metastatic disease to the thyroid may be underrecognized during life [1,13]. This discrepancy emphasizes the importance of maintaining a broad differential diagnosis when evaluating thyroid nodules in patients with a previous history of malignancy.

Our patient experienced systemic recurrence 13 years after completing treatment with curative intent, a pattern consistent with the natural history of hormone receptor-positive breast cancer. Unlike other breast cancer subtypes, luminal tumors remain at risk of recurrence for decades, with studies demonstrating an almost linear risk of relapse over time [3]. This prolonged risk has been attributed to tumor dormancy, a process in which disseminated tumor cells persist in a quiescent state while evading immune surveillance and cytotoxic therapies before becoming reactivated by changes in the hormonal, immune, or bone marrow microenvironment [14,15]. Accordingly, current NCCN recommendations support consideration of extended endocrine therapy (7.5-10 years) for selected patients with high-risk disease and, more recently, the incorporation of adjuvant CDK4/6 inhibitors in eligible patients [5]. Although these strategies were not part of the standard of care when our patient completed treatment, her clinical course reinforces the importance of long-term surveillance and sustained clinical suspicion for recurrence, even after prolonged disease-free intervals.

Surveillance imaging during follow-up identified thyroid and pulmonary nodules that ultimately led to the diagnosis of recurrent metastatic disease. The diagnostic evaluation was broadly consistent with contemporary international guidelines, including those of the ATA and NCCN, which seek to balance timely detection of recurrence with the risks of overdiagnosis and unnecessary interventions [9,16]. Nevertheless, the management of the pulmonary nodule highlights the challenges posed by small indeterminate pulmonary lesions. After enlargement of the right lower lobe nodule to 6 mm at month 12 (growth >1.5 mm), an alternative management strategy supported by the NCCN (category 2A) would have been repeat chest CT after three to six months to better assess interval growth before subsequent evaluation [16]. This case illustrates the importance of individualized surveillance strategies in high-risk patients, in whom even subtle interval growth of small pulmonary nodules may represent early metastatic disease.

The most notable pathological finding in this case was the coexistence of three histologically distinct neoplastic lesions within the same thyroid specimen: metastatic breast carcinoma, PTMC, and oncocytic adenoma (Hürthle cell adenoma). This combination represents a thyroid collision tumor, defined as the coexistence of two or more independent tumors within the same anatomical site while maintaining distinct histopathological characteristics [6].

Accurate distinction between metastatic and primary thyroid neoplasia is essential because the therapeutic approach and prognostic implications differ substantially. Ghossein et al. reported a single-center retrospective case series of 30 patients with thyroid metastases from non-thyroidal malignancies; 11 cases were consultation cases initially diagnosed at outside institutions, of which four (36%) had initially been misclassified as primary thyroid carcinomas [17]. Immunohistochemistry, interpreted in conjunction with morphology and clinical history, plays an important role in establishing the origin of thyroid lesions. Thyroid-lineage markers such as PAX8 can support a thyroid origin, although their sensitivity varies according to histologic subtype, with reported sensitivity ranging from 91% to 100% in well- and moderately differentiated thyroid carcinomas and approximately 54% in anaplastic thyroid carcinoma [18,19]. Conversely, breast-associated markers such as GATA3, together with estrogen receptor (ER) and progesterone receptor (PR) expression, may support breast origin, particularly in the appropriate clinical context [17]. However, ER and PR expression alone cannot reliably distinguish metastatic breast carcinoma from primary thyroid carcinoma, as expression of these receptors has also been reported in papillary thyroid carcinoma [20]. In the present case, the immunohistochemical profile of the thyroid lesion, including GATA3 positivity, PAX8 negativity, and ER and PR expression, supported a breast origin when interpreted together with the patient's clinical history and the morphologic findings.

Several hypotheses have been proposed to explain the occurrence of collision tumors, including a shared carcinogenic stimulus, microenvironmental changes induced by one tumor that facilitate the development of another, or simple coincidental coexistence because of the high prevalence of thyroid nodules [6]. The latter hypothesis is particularly plausible for PTMC, whose prevalence in autopsy series ranges from 1% to 35.6% [21, 22]. Supporting this possibility, in the study by Gosshein et al., 11 of 28 thyroid resection specimens (39%) showed concurrent primary thyroid neoplasms [17]. Although this finding does not establish a causal relationship between the two neoplasms, it supports the possibility that metastatic disease and incidental PTMC may coexist within the same thyroid specimen, particularly given the relatively high prevalence of occult PTMC. 

Beyond the possibility of coincidental coexistence, increasing attention has been directed toward the hypothesis of shared biological and molecular mechanisms between breast and thyroid cancer. A meta-analysis including nearly three million patients from 44 studies found that women with breast cancer have an increased standardized incidence ratio (SIR) of developing subsequent thyroid cancer (SIR 1.5, 95% confidence interval [CI] 1.3-1.7), while women with thyroid cancer similarly have an increased risk of subsequent breast cancer (SIR 1.4, 95% CI 1.2-1.6) [23]. Although these epidemiological findings do not establish causality, they support the hypothesis that the coexistence of breast and thyroid neoplasms may reflect shared biological mechanisms in at least a subset of patients. 

Estrogen signaling represents one of the most plausible molecular links between breast and thyroid neoplasia. The thyroid gland expresses both ERα and ERβ, with higher receptor expression reported in neoplastic than in normal thyroid tissue. Experimental evidence suggests that estrogen signaling promotes thyroid tumor growth and progression in animal models and human thyroid cancer cell lines, supporting a potential biological basis for the observed epidemiological association between these malignancies [24,25]. Although the present case cannot determine which of these mechanisms, if any, contributed to the observed coexistence of tumors, it highlights the biological complexity of thyroid collision tumors and reinforces the need for further investigation into the mechanisms linking breast and thyroid neoplasia.

Current NCCN guidelines recommend indefinite clinical follow-up for patients with hormone receptor-positive breast cancer, consisting of regular physical examination and annual mammography [5]. Our case raises the hypothesis that the role of targeted thyroid evaluation in selected patients at high risk of late recurrence deserves further investigation. However, current evidence does not support routine thyroid ultrasonography or additional imaging surveillance in asymptomatic patients. A Cochrane systematic review found no benefit of surveillance imaging beyond standard follow-up with respect to overall survival or quality of life, although thyroid ultrasonography was not specifically evaluated [26]. Likewise, the United States Preventive Services Task Force (USPSTF) recommends against thyroid cancer screening in the general population because of concerns regarding overdiagnosis and overtreatment [27]. Consequently, whether selected patients at high risk of late recurrence might benefit from targeted thyroid evaluation remains an unanswered research question.

The strengths of this report include long-term longitudinal follow-up through electronic health records, allowing detailed reconstruction of the patient's diagnostic evaluation, treatment, and clinical course. Histopathological examination confirmed the coexistence of metastatic breast carcinoma, PTMC, and oncocytic adenoma within the same thyroid specimen, providing detailed clinicopathological documentation of an exceptionally rare thyroid collision tumor.

The limitations are those inherent to a single case report, including limited generalizability and the inability to establish causal relationships or draw epidemiological conclusions. In addition, the observational nature of this report precludes determination of whether the coexistence of these three neoplastic lesions reflects a shared biological mechanism or a coincidental occurrence. Finally, because the patient was managed at a tertiary academic referral center, the diagnostic approach and multidisciplinary management described may not be fully applicable to other healthcare settings.

## Conclusions

This case highlights the importance of maintaining long-term clinical surveillance in patients with hormone receptor-positive breast cancer, as recurrence may occur many years after apparently curative treatment and present at unusual metastatic sites such as the thyroid. The coexistence of metastatic breast carcinoma, PTMC, and oncocytic adenoma within the same thyroid specimen illustrates the diagnostic complexity of thyroid collision tumors and underscores the importance of considering metastatic disease in the differential diagnosis of thyroid nodules in patients with a history of breast cancer. Finally, although current evidence does not support routine thyroid screening, this case generates the hypothesis that the role of targeted thyroid evaluation in selected patients at high risk of late recurrence warrants further prospective investigation.
